# Deep decarbonization of the Indian economy: 2050 prospects for wind, solar, and green hydrogen

**DOI:** 10.1016/j.isci.2022.104399

**Published:** 2022-05-13

**Authors:** Shaojie Song, Haiyang Lin, Peter Sherman, Xi Yang, Shi Chen, Xi Lu, Tianguang Lu, Xinyu Chen, Michael B. McElroy

**Affiliations:** 1 College of Environmental Science and Engineering, Nankai University, Tianjin 300350, China; 2 John A. Paulson School of Engineering and Applied Sciences, Harvard University, Cambridge, MA 02138, USA; 3 Institute of Thermal Science and Technology, Shandong University, Jinan 250061, China; 4 Department of Earth and Planetary Sciences, Harvard University, Cambridge, MA 02138, USA; 5 School of Environment, State Key Joint Laboratory of Environment Simulation and Pollution Control, Tsinghua University, Beijing 100084, China; 6 School of Electrical Engineering, Shandong University, Jinan 250061, China; 7 School of Electrical and Electronic Engineering, Huazhong University of Science and Technology, Wuhan 430074, China

**Keywords:** Energy resources, Energy policy, Energy sustainability, Energy management, Energy Modeling, Energy Systems

## Abstract

The paper explores options for a 2050 carbon free energy future for India. Onshore wind and solar sources are projected as the dominant primary contributions to this objective. The analysis envisages an important role for so-called green hydrogen produced by electrolysis fueled by these carbon free energy sources. This hydrogen source can be used to accommodate for the intrinsic variability of wind and solar complementing opportunities for storage of power by batteries and pumped hydro. The green source of hydrogen can be used also to supplant current industrial uses of gray hydrogen produced in the Indian context largely from natural gas with important related emissions of CO_2_. The paper explores further options for use of green hydrogen to lower emissions from otherwise difficult to abate sectors of both industry and transport. The analysis is applied to identify the least cost options to meet India’s zero carbon future.

## Highlights

• A novel integrated renewable energy-hydrogen planning model is developed for India
• Scenarios that include green H_2_ could reduce 2050 energy system costs by 10%
• Green hydrogen allows for cheap decarbonization of industrial and power sectors

## Graphical abstract

**Figure:**
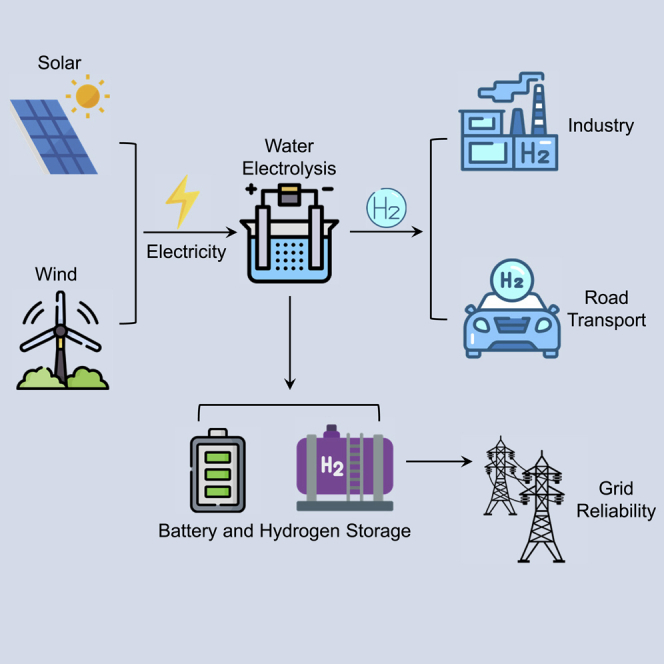

## Introduction

India is currently the world’s third largest national CO_2_ emitter, accounting for 7% of the global total, although its per capita emissions amount to only 40% of the global average (Friedlingstein et al., 2020; International Energy Agency, 2021). Coal is responsible for 45% of India’s contemporary total energy consumption, followed by petroleum and natural gas, 32%, with traditional biomass and organic waste accounting for an additional 20%. Other renewable sources, including solar, wind, and hydroelectricity, account for only about 2% of total primary energy consumption (U.S. Energy Information Administration, 2020). According to India’s Central Electricity Authority, the country had more than 370 GW of installed utility-based electricity generating capacity connected to its national grid in June 2020, dominated mainly by coal (55%). Renewable energy accounted for a lower share (12% for large hydropower projects and almost 24% for other renewables) but has grown significantly over the past several years (Ministry of New and Renewable Energy of India, 2020).

India has announced a 2070 net-zero emissions target at the most recent United Nations Climate Change Conference in Glasgow (Climate Action Tracker, 2021). However, the Glasgow Climate Pact highlighted that limiting global warming to 1.5°C above preindustrial levels would require reduction of global carbon emissions to net-zero by around 2050 (O’Grady, 2021). A global 2070 net-zero carbon emission pathway corresponds to global warming of 2°C above preindustrial levels and means higher climate-related risks for natural and human systems (IPCC, 2018). Therefore, it is meaningful to investigate India’s net-zero emission scenario for 2050 and to identify the contribution of renewable energy in such a scenario.

A report by The Energy and Resources Institute (TERI) and Shell (referred to subsequently as the TERI/Shell report) discussed options for future development of the Indian energy system in the context of ongoing projections for the evolution of the country’s economy (TERI/Shell, 2021). The report identified a path to steer the domestic energy system toward net-zero emissions by 2050, while achieving India’s sustainable economic development ambitions. To reach a net-zero emissions energy system by 2050, India will need to deploy clean energy technologies on a massive scale. It will require more and faster deployment of large-scale solar, wind, and hydro power — replacing coal — to power greater electrification across the country. It will also require the development of new fuels, such as liquid biofuels and biogas. In addition, carbon removals (from technology and nature) will likely be required to play an additional critical role. There have been recent studies also investigating the high penetration of renewable energy in the electricity system with low economic costs including reports by Deshmukh et al. (2021) and Gulagi et al. (2017). In this paper, we further investigate the potentially important changes in the costs and structure of India’s energy system resulting from the application of renewable hydrogen.

Electrification of energy services in combination with increased use of renewables (notably solar and wind) in electricity generation has been considered a critical element in the path to replace fossil fuels (Williams et al., 2012). Lu et al. (2020) suggested that an investment in renewables at a level consistent with meeting 80% of projected 2040 power demand could result in a lower electricity cost together with a significant reduction in emissions of CO_2_ compared to what might be expected if the power sector were to continue to rely on its current coal dominated trajectory. Rudnick et al. (2022) highlighted the key role of renewable energy technologies, in particular solar PV, for the 2040 Indian electricity sector. However, several key industrial processes and demands for long distance transport face cost, technology, and practical barriers to full-scale electrification (Henry et al., 2020). We shall argue here that use of so-called “green hydrogen” — made from the electrolysis of water powered by renewables — can offer an efficient means to effectively decarbonize many of these hard to abate sectors (van Renssen, 2020).

Focusing on the decarbonization of all sectors in India, for the first time, this paper considers multiple potential applications of green hydrogen associated with a 100% renewable power-based electricity system targeted to support a carbon-neutral economy for India by 2050. A novel integrated renewable energy-hydrogen planning model is developed to identify the least cost option to meet this objective. The model incorporates analysis of potentially available renewable resources, together with a forecast of future energy demand defining a strategy for optimal design and operation of a 100% renewable power-green hydrogen energy system.

In this study, we shall argue that green hydrogen can play an important role as a storage medium to address the inherent variability of solar and wind power generation. Surplus electricity can be used to produce hydrogen through water electrolysis. This hydrogen can be stored and made available subsequently to produce power when sources from sustainable generation, primarily wind and solar, are temporarily unable to keep up with demand. In addition to its application in the electricity sector, the analysis that follows suggests that green hydrogen can be employed to phase out the current fossil fuel derived demand for hydrogen in the industrial sector, with further opportunities to decarbonize hard-to-abate industries, with additional contributions to meet the demand for power in elements of the transportation sector. Specifically, hydrogen can be employed as a substitute for coal in the iron and steel industry as a heat source for various industrial applications and as fuel for long distance truck and bus transport.

Current sources of hydrogen are not green. Hydrogen is produced primarily from either natural gas or coal, mainly natural gas in most regions of the world with an important contribution from coal in China. In India, hydrogen is used mainly in the oil refinery sector and in the production of nitrogen fertilizer. The annual hydrogen demand for these two sectors amounted to 4.36 megatons (Mt) in 2019 and is projected to grow to 137 Mt in 2050 considering future expansion. Hydrogen in India is produced mainly from natural gas and is classified consequently as gray rather than green. Production of 1 kg of gray hydrogen is associated with the emission of about 10 kg of CO_2_. The Indian government implemented a policy in 2015 to ensure uninterrupted supply of natural gas to urea manufacturing plants (Ministry of Petroleum and Natural Gas, 2015). The fertilizer industry, India’s largest consumer of natural gas, was responsible for 12.9 Mt of natural gas consumption in 2018, accounting for 28% of total national consumption (Ministry of Petroleum and Natural Gas, 2020). According to the International Energy Agency (IEA) (International Energy Agency, 2019), the cost for gray hydrogen in India is about $1.8 kg^−1^. In what follows, we shall argue that a significant quantity of green hydrogen could be made available in India at costs competitive with current production from natural gas.

In this study, four model scenarios are explored for the year 2050. In the baseline (S1_Base), the energy supply is dominated by fossil fuels. The capacity for renewable power is set at 450 GW for 2050, consistent with the 2030 target announced by the Indian government (Government of India, 2016; Press Trust of India, 2020). The implicit assumption in S1_Base is that India will not increase renewable energy capacity beyond 2030. The share of electricity in the energy system is projected to increase significantly in the transportation sector (excluding long-distance trucks), in agriculture (pumping and tractors), and in the commercial and residential sectors. In a second scenario, identified as the variable renewable penetration (VRP) option, we explore the costs for different assumed levels of penetration for renewables (i.e., 40%, 60%, 80%, and 100%) required to satisfy the same amount of expanded electricity demand identified in the baseline scenario. Scenarios 3 and 4 assume that renewable sources account for 100% of power production addressing specifically opportunities and benefits for integration of renewable (green) hydrogen. The hydrogen in scenario 3 (S3_GH_2_P) is deployed in only the electricity sector, providing an opportunity to compensate for shortfalls that would arise otherwise in meeting the demand for power from what would amount otherwise to an intrinsically variable source. Scenario 4 (S4_GH_2_All) explores a more expansive application of green hydrogen, with deployment to facilitate decarbonization of hard-to-abate sectors in both industry and transport, at the same time eliminating totally the current industrial dependence on gray hydrogen. For all four scenarios, we use an integrated optimal planning model to identify related least costs and associated CO_2_ emissions.

## Results and discussion

### Projected hourly electricity demands

According to the NITI prediction (National Institution for Transforming India, 2017), in 2047, India’s electricity demand under the BAU scenario is 5,651 TWh and that with the less ambitious scenario is 5,144 TWh. TERI also predicted the electricity demand for India in 2051 (TERI/Shell, 2021). In the reference energy scenario, the electricity demand is 7,607 TWh; in the 100% renewable energy scenario, it is 8,108 TWh. Our analysis shows that the annual countrywide electricity demand is projected to grow from 947 TWh in 2015 to 8,323 TWh by 2050. The prediction in 2050 is consistent with TERI’s study and higher than NITI’s prediction. Globally, IEA forecasts the electricity demand for China, the United States, and Europe in 2050 based on three scenarios (International Energy Agency, 2021). In their “sustainable development” scenario, electricity demands are 7,435 TWh for the US, 7,267 TWh for Europe and 15,329 TWh for China. Compared with other countries, India’s 8,323 TWh predicted electricity demand is higher than the electricity demands of the US and Europe, and around half of China’s electricity demand for 2050 (International Energy Agency, 2021).

**Figure 1:**
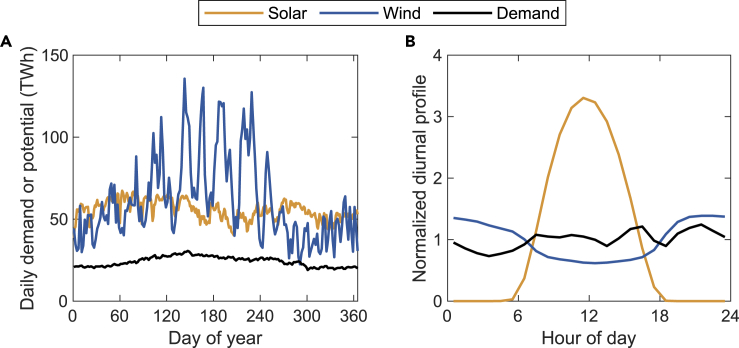
National solar and wind power potential and 2050 demand (A) Daily variability (TWh); (B) yearly average normalized diurnal profile. The diurnal profiles are normalized (y axis unitless) by the 24-h average values.

Estimates for the 2050 projected hourly demands for electricity consistent with S1_Base and S2_100%RP are presented in Figure 1. The procedures used to derive these estimates are described in the STAR Methods section. Data for individual regions are displayed in Figure S1. Figures 1 and S1 also include estimates for the physical potential for production of electricity from wind and solar. Assumptions used in developing these data are described in STAR Methods.

The demand for electricity exhibits variability on both diurnal and seasonal time scales. On the seasonal scale, requirements typically peak in summer, attributable both to the intrinsically higher demands for electricity in that season and amplified by increased requirements for air conditioning (AC). The demand pattern corresponds generally well with the physical potential for wind, which also peaks during summer responding to the higher wind conditions associated with the summer monsoon. In contrast, the analysis indicates a minimum in the potential for solar generated power in summer, reflecting a combination of higher temperatures (which reduce the efficiency of solar panels) and cloudier conditions because of the monsoon. Given the pronounced diurnal variability in the solar and to a lesser extent the wind resource, it is clear that if renewables are to provide the dominant future source of electric power, important investments will be needed in storage and transmission facilities to address anticipated shortfalls in supplies of power particularly during evenings in summer. That being said, as indicated in Figures 1 and S1, there are abundant potential resources for both solar and wind, which suggests that the combination can make a major contribution to India’s future path to ultimate decarbonization. On the local level, there is significant overlap between zones of highest demand and regions of largest renewable potential, particularly in the South and West regions (Figure S1). Production and storage of green hydrogen and all components of India’s electricity system involving wind, solar, hydro, coal, gas, storage, and interregional transmission are optimally configured and operated to meet these spatially distributed and temporally defined demands for power and hydrogen. To meet the power demand on an hourly basis at the regional level, we explore these inputs further using our integrated renewable energy-hydrogen planning model.

### Scenario results from the integrated planning model

**Figure 2:**
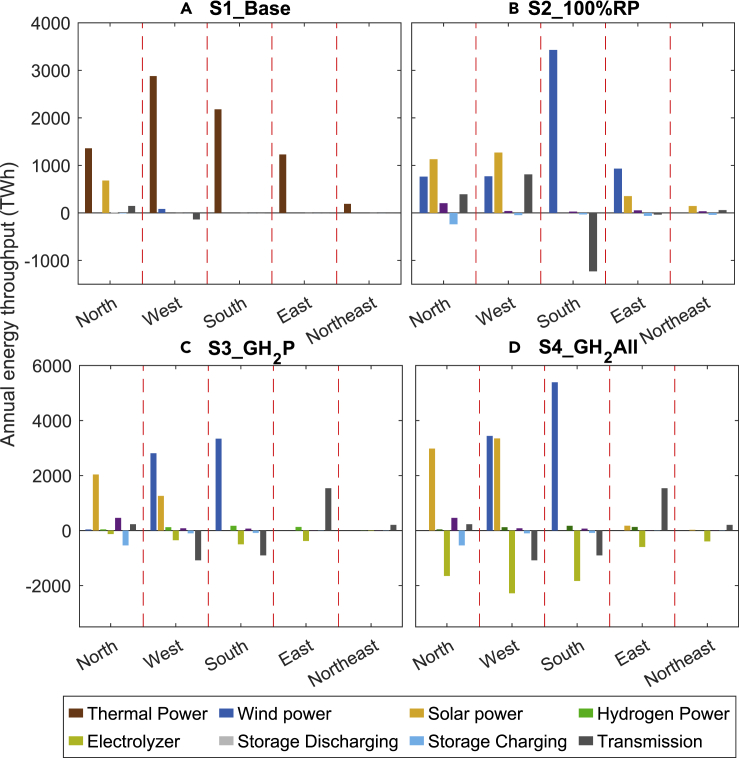
Regional energy throughput distribution Regional distribution of energy throughput (TWh) for different technologies for 2050 inferred using the optimization model. Curtailed solar and wind power are not included in the figure. Data identified as transmission refer to interregional transfers of power over the transmission network. Negative transmission values identify exports; positive values imports.

Regional distributions of electricity generation and export for different technologies inferred with the model for the 2050 energy supply are summarized in Figure 2. The West, where the highest electricity and hydrogen demand are concentrated, has a large volume of energy throughput; close to 30% of the electricity consumed in the West is transferred from the South in S2_100%RP. In S1_Base, 416 GW of solar capacity is located in the North. This represents a large apportionment compared with capacities installed in other regions under this scenario, reflecting the relatively low levelized costs for solar in the North. In scenarios 2-4, wind (in the South and West) and solar (North and West) are both significant sources of electricity, consistent with the renewable resources available in these regions (Figure S1). Under the hydrogen pathways (scenarios 3 and 4), the bulk of the electricity consumed in the East and Northeast is derived from other regions and used, in large part, for electrolysis. Notably, the overall electricity generated from wind is much larger relative to that from solar power, reflecting the larger capacity factors associated with the former (indicated also in Figure S2).

**Figure 3:**
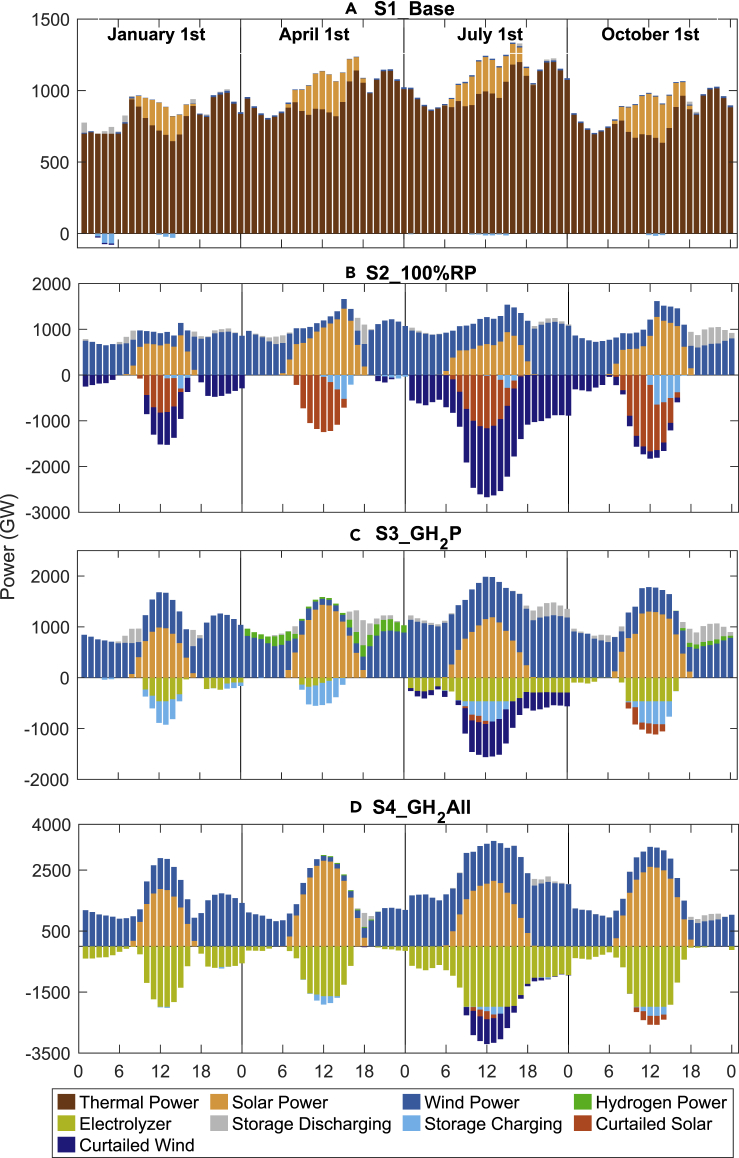
Seasonal hourly power flow Countrywide hourly power flows (GW) defined for seasonally representative days in 2050 based on the model simulation for the selected four scenarios. Positive values refer to power generation; negative values refer to storage of power, curtailed electricity and use of power to produce hydrogen.

The diurnal and seasonal patterns for solar and wind power can have an important impact on hourly system operations throughout the year, particularly for the scenarios featuring large capacities of renewables. The 2050 hourly power balance for a typical day for each season is displayed for each of the selected scenarios in Figure 3. In S1_Base, thermal power remains the predominant source of India’s electricity in all seasons, with relatively minor contributions (8.7%) from wind and solar. Under S2_100%RP, wind and solar emerge as the predominant sources of electricity, with solar a significant contributor in the afternoons with wind playing a major role throughout the day, particularly in the evenings. However, with the adoption of these two intermittent sources of electricity without compatible storage options, large amounts of both wind and solar generated electricity are curtailed, most notably during the monsoon season when curtailment could account for more than 50% of the potential source. This underscores the importance for the integration of hydrogen technologies in scenarios 3 and 4, which allows for much of the curtailed electricity to be used for electrolysis of water rather than wasted (as seen in Figures 2C and 2D). It is noted that hydrogen power is generated mainly in transitional seasons (spring and autumn) compensating for the shortage of renewable generation during morning and evening hours (as indicated by green bars in Figure 3C). The national average annual renewable power curtailment rate is reduced from 35.7% under S2_100%RP to 7.3% and 3.3% in the scenarios which include hydrogen, S3_GH_2_P and S4_GH_2_All, respectively.

**Figure 4:**
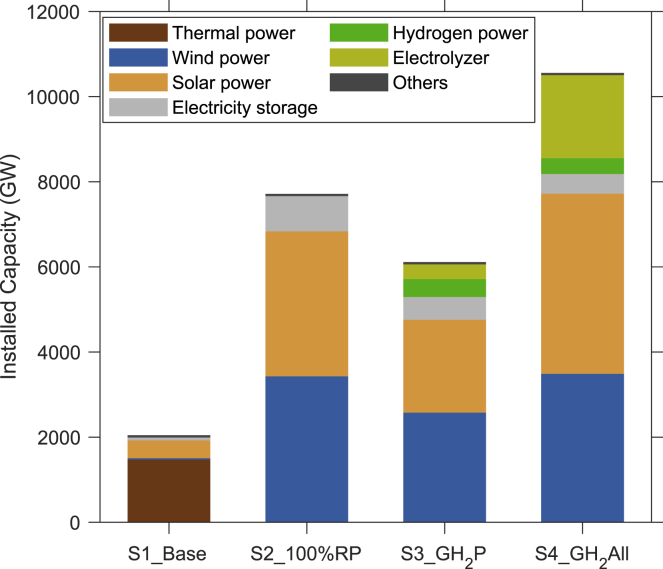
National 2050 installed capacities for different technologies Countrywide installed capacities (GW) for different technologies identified using the optimization model with parameters appropriate for the selected 4 cases. Others represent the capacities for hydro and nuclear power, which are assumed to be the same as the current situation.

As indicated by the national capacities for the four scenarios as summarized in Figure 4, the scenarios with hydrogen applications (S3_GH_2_P and S4_GH_2_All) have fewer requirements for electricity storage, because green hydrogen plays an important role as a storage medium, addressing the inherent variability of renewable power. Notably, hydrogen operates as a long term (seasonal) storage mechanism to increase the overall utilization rate of renewable power, while batteries can store energy only for more limited time intervals (as indicated by the SOC variation in Figure S3 versus Figure S4). Consequently, the utilization of battery systems improves significantly under S3_GH_2_P compared to that under S2_100%RP, the annual output per installed capacity (MWh) of battery in the two scenarios is 298 MWh and 89 MWh, respectively. Even though the annual output from hydrogen to power systems is relatively lower compared to the battery source (Figure S5), it’s vital for operational safety to have storage capable of supplying power for the longer term. Hydrogen to power systems once operated continuously in the model for more than two weeks during low renewable power periods. With the inclusion of green hydrogen to meet demand for the power sector (S3_GH_2_P), the national installed capacities for both solar and wind power are lowered significantly compared to the estimates in the 100% renewable power scenario (S2_100%RP). However, compared to the large amount of hydrogen consumed by industry and the transport sector (totaling 136.7 million metric tons in 2050) under S4_GH_2_All, the amount of hydrogen (roughly 4 million metric tons) used for power generation is much less. Annual electricity consumption (excluding curtailment) increases from 8800 TWh under S2_100%RP to 9,578 TWh and 15,483 TWh under S3_GH_2_P and S4_GH_2_All, respectively. Larger installed capacities of renewables are required for a deep decarbonization pathway. This potential source for green hydrogen is used also to reduce system costs and emissions.

**Figure 5:**
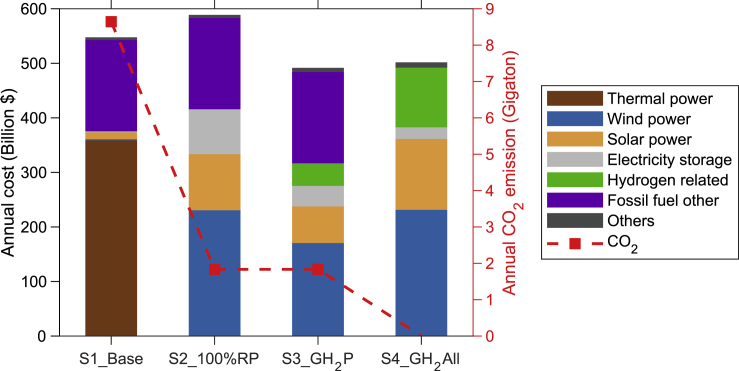
National energy supply cost Annual costs (Billion $) for the 2050 energy system estimated for the selected four cases. “Thermal power” refers to the costs for fuel, amortized capital, operational and maintenance expenses for coal and gas-fired plants. “Hydrogen related” refers to the costs associated with capital and operational expenses for electrolyzers, hydrogen turbine, compressor, and hydrogen storage. “Fossil fuel other” refers to the costs for coal, oil, and natural gas consumed by selected sectors excluding power generation. “Others” refer to the costs for amortized capital and operational and maintenance expenses for hydro and nuclear plants. The red dashed line indicates annual CO_2_ emissions (Gt) associated with each scenario.

Costs and CO_2_ emissions associated with the various scenarios derived from the integrated systems model are indicated for 2050 in Figure 5. The system costs for S1_Base are high – exceeding $500 billion – and are surpassed only by results obtained with the 100% renewable scenario. S2_100%RP is particularly costly as significant capacities for wind, solar and electricity storage are required in order to deal with intermittencies in the source of renewables. At lower levels of commitment to renewables (40%–80%), the annual costs associated with the generation mix are less than the costs identified for S1_Base, reflecting the fact that the levelized costs for wind and solar power are much lower than costs for fuel and operational expenses associated with thermal power (Figure S6). Although the renewable power scenarios studied here can contribute to a reduction in emissions of CO_2_ from the power sector, emissions from other sectors – particularly long-range transport and industrial uses – would remain unabated. The scenarios with hydrogen penetration (S3_GH_2_P and S4_GH_2_All) can reduce the overall system costs relative to S1_Base, with the ancillary benefit of significantly curtailing CO_2_ emissions. Compared to S3_GH_2_P, the marginal cost of hydrogen supply for industry and transport in S4_GH_2_All is roughly 1.3 $ kg^−1^, representing a relatively low CO_2_ abatement cost for the hard to abate sectors at about 5.1 $ ton^−1^ (with an average value at 1.7 $ ton^−1^ for all the sensitivity scenarios). Compared to S1_Base, a negative CO_2_ abatement cost, −5.3 $ ton^−1^, is realized under S4_GH_2_All. This suggests that absent a significant investment in green hydrogen, a 100% renewable power sector may be less economical.

To estimate uncertainty bounds for the studied decarbonization pathways, national expense results for the sensitivity scenarios considering a range of costs for various technologies, poorer wind resource quality and possible expansions on hydropower and nuclear power are presented in Table 1. Compared to the moderate trend, these different scenarios consider ±20% cost variations for renewables (including the wind and solar power systems, electricity storage and transmission), fossil fuels (including thermal power plant and fuel price) and hydrogen technologies (including electrolysis, hydrogen compression, delivery, storage, and power generation). We consider a scenario where the hourly capacity factors for wind power decrease by 10%, representing a lower availability of wind resource, consequently scaling down the national annual average capacity factor from 22.7% to 20.4%. With another scenario using the meteorological data for the year of 2020 to account for changes in both total output and tempo-spatial variability in wind resources, the estimated national annual average capacity factor is roughly 7% lower than that of the base year. Accounting for potential high hydro and nuclear buildout in the future, we investigate another scenario which assumes that the capacities of hydro and nuclear power could expand to 73,445 MW and 16,880 MW respectively, following the scenario from Deshmukh et al. (2021). It is found that an expansion of hydro and nuclear power is likely to reduce the overall energy supply cost in 2050. As for hydrogen implementations – with the exception of the low fossil fuel cost scenario – the national expenses with hydrogen application for deep decarbonization (S4_GH2All) are always lower than that for fossil fuel dominated alternatives (S1_Base). Meanwhile, a 100% renewable power sector without the application of long-term storage (S2_100%RP) is the most expensive of all the sensitivity scenarios, indicating the importance of practical hydrogen applications in reducing electricity costs and associated emissions. Although wind variability of an alternative year is taken into account, the simulated results are consistent with that from other scenarios, which ensures the robustness of the main findings. Capacity breakdown is provided in Figure S7 reflecting also the economic competitiveness and optimal combination of the studied technologies.

**Table 1:** National expense of sensitivity scenarios (Billion $)

	S1 Base	S2 40%RP	S2 60%RP	S2 80%RP	S2 100%RP	S3 GH2P	S4 GH2All
Moderate trend	548	504	489	482	589	493	502
Renewable high cost	551	522	518	525	673	580	549
Renewable low cost	544	485	459	439	508	436	424
Fossil fuel high cost	653	585	556	535	622	527	/
Fossil fuel low cost	442	422	421	429	555	460	/
Hydrogen high cost	/	/	/	/	/	496	527
Hydrogen low cost	/	/	/	/	/	489	477
Wind low availability	548	509	499	496	613	512	529
Wind alternate year	548	507	496	493	623	511	528
High hydro and nuclear	544	500	486	479	583	490	499

The moderate trend represents the main scenarios presented in the study. High and low cost scenarios indicate +20% and −20% cost variation compared to the moderate trend scenarios. Wind low availability scenario accounts for a 10% decrease in wind power capacity factors relative to the moderate trend scenarios. Wind alternate year scenario takes the MERRA-2 data for year 2020 accounting for changes in both total output and tempo-spatial variability in wind resources. The high hydro and nuclear scenario indicates expanded capacity for hydro and nuclear power.

### Conclusions

The paper sought to identify a least cost option to meet a targeted carbon neutral scenario for India by 2050. It placed particular emphasis on wind and solar sources of electricity complemented by applications of green hydrogen both as a means to compensate for the intrinsic variability of these sources, with further deployments in difficult to abate sectors of both industry and transport. The integration of hydrogen technologies allows for much of the curtailed electricity to be used for electrolysis of water rather than wasted, thereby reducing the national average annual renewable power curtailment rate from 35.7% to 3.3%. The scenarios with hydrogen penetration can also reduce the overall system costs relative to base scenario, with the ancillary benefit of significantly curtailing carbon emissions, therefore realizing a negative abatement cost of about −5.3 $ per ton CO_2_.

### Limitations of the study

There are a few caveats that should be noted. (*i*) We acknowledge that the current stage of India’s grid dispatching is far away from a national-level optimal dispatch. We anticipate and assume a target for future system expansion assuming a much better grid structure in 2050. (*ii*) We recognize that the option developed here is certainly not unique. Further studies could pay attention to other low carbon possibilities including for example investments in recycling and utilization of India’s extensive sources of not only urban but also rural organic waste, much of which is currently incinerated with serious negative impacts not only on air quality but also for public health. (*iii*) Several sectors were not explicitly considered in the present study including, for example, aviation and shipping. We anticipate that the carbon sink created through investments in forest and tree cover could compensate for most of the emissions from these sectors. We believe that products derived at least in part from green hydrogen would also contribute usefully to both these applications, a topic, however, beyond the scope of the present study, to be explored potentially in a follow up study.

## STAR★Methods

### Key resources table

**Table:** 

REAGENT or RESOURCE	SOURCE	IDENTIFIER
**Software and algorithms**		

MATLAB R2021b	The MathWorks, Inc.	https://matlab.mathworks.com/
Gurobi 9.1.0	Gurobi Optimization, LLC.	https://www.gurobi.com/
YALMIP	Johan Löfberg	https://yalmip.github.io/
GAMS	GAMS Development Corp.	https://www.gams.com/

**Other**		

Socioeconomic Indicators	International Energy Agency; United Nations Development Program	https://www.iea.org/reports/india-energy-outlook-2021 https://population.un.org/wpp/Publications/Files/WPP2019_Highlights.pdf
Road transport sector	Multiple sources	https://morth.nic.in/road-transport-year-book https://www.iea.org/reports/global-ev-outlook-2020 https://doi.org/10.1016/j.enpol.2018.01.037 https://doi.org/10.1016/j.atmosenv.2018.02.053
Industrial energy efficiency	Copenhagen Center on Energy Efficiency	https://c2e2.unepdtu.org/wp-content/uploads/sites/3/2017/06/enhancing-energy-efficiency-in-india-assessment-of-sectoral-potentials.pdf
Hourly electricity demand profile	Power System Operation Corporation	https://power.carboncopy.info/
Hourly temperature profile	NASA reanalysis product	https://gmao.gsfc.nasa.gov/reanalysis/
Electric vehicle charging profile	National Renewable Energy Laboratory	https://www.osti.gov/biblio/1659823
Wind resource	NASA reanalysis product	https://gmao.gsfc.nasa.gov/reanalysis/
Solar resource	NASA weather analysis product	https://gmao.gsfc.nasa.gov/weather_prediction/
Land cover and slope	NASA MODIS land cover product; Shuttle Radar Topography Mission	https://lpdaac.usgs.gov/dataset_discovery/modis/modis_products_table/mcd12c1/ http://srtm.csi.cgiar.org

Wind turbine	Goldwind	https://www.goldwindamericas.com/
Solar PV panel	Multiple sources	https://doi.org/10.1016/j.joule.2019.06.006
Water electrolysis	International Energy Agency	www.iea.org/reports/the-future-of-hydrogen
Hydrogen storage and delivery	US Department of Energy	https://www.energy.gov/eere/fuelcells/articles/hydrogen-and-fuel-cell-technologies-office-multi-year-research-development
Solar and wind power	International Renewable Energy Agency; National Renewable Energy Laboratory	https://www.irena.org/-/media/Files/IRENA/Agency/Publication/2020/Jun/IRENA_Power_Generation_Costs_2019.pdf https://atb.nrel.gov/
Pumped hydro and battery systems	US Department of Energy; National Renewable Energy Laboratory	https://energystorage.pnnl.gov/pdf/PNNL-28866.pdf https://www.nrel.gov/docs/fy20osti/75385.pdf
Carbon emission factor	India Ministry of Power	https://cea.nic.in/cdm-co2-baseline-database/?lang=en
Inter-regional transmission	India Ministry of Power	https://cea.nic.in/annual-report/?lang=en

### Resource availability

#### Lead contact

Further information and requests for resources should be directed to and will be fulfilled by the lead contact, Dr. Michael B. McElroy (mbm@seas.harvard.edu).

#### Materials availability

This study did not generate new materials.

### Method details

#### Future energy service demand

The methods used to predict India’s future final energy demand involve econometric regression analysis and elasticity coefficient analysis. Prediction of the final energy service demand varies by sectors with different drivers, which include final transport service demand, industrial products service demand, agriculture service demand, and residential and commercial service demands. As introduced earlier, several sectors were not considered in this study specifically including aviation and shipping. We chose 2015 as the base year and obtained data on energy consumption for different sectors from various statistical yearbooks and annual reports for India.

Trends in GDP, population, and urbanization are the key drivers determining future demands for energy in the Indian economy. NITI Aayog (The National Institute for Transforming India), the Indian government entity responsible for drafting recommendations for policy, projected that India’s GDP could maintain an annual growth rate as high as 8.5% out to 2042, decreasing subsequently to 7.4% over the period 2042‒2047 (National Institution for Transforming India, 2017). For present purposes, we assume that the trend in GDP post-2022 transitions steadily back to the level prior to COVID-19 (International Energy Agency, 2021). We assume growth rates of 6.0% for 2022‒2030, 6.5% for 2030‒2040, and 5.7% for 2040‒2050, consistent with the recommendations from the International Energy Agency (IEA), the United Nations Development Program (UNDP), and the World Bank. Our assumption is consistent with the TERI/Shell projection that the Indian economy can resume a rapid recovery realizing a national GDP of $5 trillion by 2025 (TERI/Shell, 2021). The population of India is currently the second largest in the world trailing only China. The assumptions adopted in this study with respect to trends in future Indian population and patterns of urbanization, consistent with the studies by UNDP, IEA, and TERI, are summarized for the coming decades in Table S1. UNDP suggests that the Indian population will rise from 1.37 billion people in 2019 to 1.6 billion in 2040 surpassing China as the world’s most populous country by 2025 (United Nations, 2019). The UN conclusions are consistent with estimates by NITI projecting an increase in the population of India to a level of 1.68 billion by 2047.

The final energy service demand in the transport sector is broken down in terms of passenger and freight turnover. The controlling factors for passenger transportation service are per capita income, and responses to increasing trends in urbanization and population. For freight demand, the driving factors are income, urbanization, and GDP growth. The base year vehicle stock for road transport is taken from the India Statistical Yearbook (Ministry of Road Transport and Highways, 2018a, 2018b). Data on the fuel economy, mileage per vehicle, and load factors are obtained from a number of sources (International Energy Agency, 2019, 2020; Paladugula et al., 2018; Prakash and Habib, 2018). Components of the evolving road transportation system are summarized in Table S2, with final energy service demand presented in Table S3. The current railway system in India depends largely on fossil fuel as an energy source, and we assume a significant transition to electricity by 2050.

India’s residential final energy service demand includes heating, cooling, lighting, hot water, cooking, and residential appliances. The energy consumed currently to meet the residential service demand involves a combination of fossil fuels and electricity. Comparatively, the final demand for commercial energy does not include cooking and hot water, and electricity is consumed primarily for air conditioning and commercial appliances. Since there is a lack of data for predictions on the allocation of future absolute area spaces for India, we consider as driving factors population, rate of urbanization, and GDP growth. Demand for air conditioning will be particularly important given the frequency of hot and humid conditions experienced already in India in summer, a problem likely to become more serious in a future warmer climate (International Energy Agency, 2018). The treatment of requirements for energy in the agricultural sector is simplified in this study, assumed to be driven primarily by growth in GDP. The current energy consumption involves a combination of electricity and fossil fuels (mainly diesel). As shown in Table S4, the final energy service demands in the residential, commercial and agricultural sectors are expected to increase by a factor of 2‒4 over the period 2020‒2050.

In the industrial sector, the predictions vary for different products. Demands for key products, including cement, iron & steel, fertilizer, aluminum, caustic soda, soda, paper, and textile, are analyzed out to 2050 (Table S5). Interactions among sectors are considered. Taking iron and steel as an example, demand for iron and steel products involves mainly three components: residential and commercial buildings, household appliances, and vehicles in the transport sector. Thus, iron and steel demands in this study are aggregated in terms of requirements for buildings driven by population and GDP, household appliances driven by population and incomes, and demands for the transport sector driven by population and growth in GDP. The demands for iron and steel predicted in our study correspond to 222.5 Mt in 2030 rising to 364.6 Mt in 2050, consistent with data from the NITI and UNEP studies (National Institution for Transforming India, 2017; Vishwanathan et al., 2017). Data on energy intensity and improvements in energy efficiency over time are adopted primarily from Vishwanathan et al. (2017).

The annual country-wide demand for electricity is projected to grow from 947 TWh in 2015–8323 TWh by 2050. Contributions to the time evolving demand out to 2050 are summarized in Table S6. The electrification rates for the residential, commercial, and agricultural sectors are taken as 100% in 2050. Together these three sectors contribute about 60% of total electricity demand projected for 2050. The electrification rate for the industrial sector is taken as about 34% in 2050, consistent with the TERI/Shell (2021). The bulk of the energy demand for the industrial sector will be satisfied in 2050 by either fossil fuels or hydrogen depending on the model scenarios. We assume, in 2050, the light vehicles and trains are fueled 100% by electric power, and that the long-distance road transport (buses and trucks) relies on either oil or hydrogen depending on model scenarios.

#### Future hourly electricity demand

To define the 2050 temporal profile for regional electricity, we estimated the hourly demand for cooling, heating and transportation and used these data to adjust and extrapolate hourly demands observed in 2019 for the five regions considered in this study. The data on regional hourly demand for 2019 were obtained from the Power System Operation Corporation under India’s Ministry of Power (Carboncopy.info, 2021). Daily and hourly demands for electricity for the target year selected for this study are summarized in Figure 1.

The total electricity demand for cooling and heating is defined for each hour of the year taking account of the geographical distribution of population and hourly variations of ambient temperature. First, temperatures for each hour of 2019 were derived from the Modern-Era Retrospective Analysis for Research and Applications, Version 2 (MERRA-2) (Gelaro et al., 2017) and were applied to calculate cooling degree-days (CDDs) and heating degree-days (HDDs) for each region. Hourly variations of temperature with time in 2050 are assumed to follow the pattern observed in 2019. The CDDs and HDDs were then weighted spatially in terms of population densities reported in India’s 2011 census (Chandramouli and General, 2011). The computed population-weighted CDDs and HDDs represent the regional daily shares estimated for the annual cooling and heating demand. Finally, the daily demands are downscaled to hourly resolution according to representative daily demand profiles (Parker, 2003). The total transportation electricity demand was allocated first to each region according to the current geographical distribution of registered vehicles (Ministry of Road Transport and Highways, 2018a, 2018b), downscaled then to hourly demand ignoring potential variations in daily shares. The representative daily charging profile for electric vehicles was obtained from the Greening the Grid (GTG) project conducted by India’s Ministry of Power (MOP) and the National Renewable Energy Laboratory (NREL) (Nagarajan et al., 2020).

#### Future energy supply

Four scenarios are identified to explore India’s energy system in 2050 covering five regions (East, North, South, West, and Northeast), accounting for regionally distributed demand, operational properties of power plants (thermal, hydro, and nuclear), potentials for renewables (solar and wind), opportunities for hydrogen production, and the availability of systems for storage and for exchange of power over the inter-regional power grid.

Unit information (hydro and nuclear) and capacities of inter-regional transmission corridors were derived from India’s Ministry of Power (2020). Capacities for nuclear and hydropower units were fixed. We assumed that there would be no further expansion of these facilities prior to 2050. We considered as an important operational component of India’s energy system the option for capacity expansion of the existing seven inter-regional transmission corridors. We assumed that the number and location of corridors are fixed but that the transmission capacity for a given corridor could be expanded according to an expansion factor, defined as the ratio of total investment cost for a corridor to its capacity (Table S7). In addition to hydrogen, two options, pumped hydro and battery systems, were considered for storage of power. Costs and characteristics of different systems, adopted from the DOE study (Mongird et al., 2019) and the NREL estimation (Cole and Frazier, 2020), are summarized in Table S8.

Cost reductions from 2020 to 2050 for renewable power systems, thermal plants, transmission lines and storage are assumed to follow the moderate trend identified in the NREL ATB database (National Renewable Energy Laboratory, 2020). The costs for coal and natural gas in 2050 are taken as $3.6 per MMBTU and $7.65 per MMBTU, respectively, consistent with the assumptions in Lu et al. (2020). Oil costs for the same year are estimated at $70 per barrel. All the costs quoted here are defined in terms of 2015 US dollars. Emission factors for coal and natural gas fired units were adopted from the Ministry of Power (Sharma et al., 2018).

#### Renewable energy potentials and costs

The wind data used in the study were derived based on year 2019 input from MERRA-2, a NASA reanalysis product publicly available from NASA’s Goddard Earth Sciences Data and Information Services Center (Gelaro et al., 2017). This database defines hourly wind speeds with a spatial resolution of 0.50° longitude by 0.67° latitude from 1980 to present. Wind speeds at 100 m were extrapolated from data at 10 and 50 m using the vertical profile of the power law described by Archer and Jacobson (2005). The friction coefficient in the analysis was evaluated using wind speeds represented at 10 and 50 m for each grid cell, as in Lu et al. (2013). Onshore wind power was computed on an hourly basis using the power curve for the Goldwind 2.5 MW turbine (Table S9). The potential for offshore wind is small in India (given the relatively large depth of waters in the country’s near shore coastal environment); so we chose in this study to exclude that option. The solar data used here were derived from NASA’s GEOS-5 FP database (Lucchesi, 2013), which identifies hourly temperatures and incident solar radiation at a spatial resolution of 0.25° latitude by 0.31° longitude. We employed an integrated solar PV assessment model in evaluating the performance of solar PV systems, following the approach described by Chen et al. (2019). Capacity factors (CFs), defined by the ratio of electricity generated by a given technology relative to the realization of its full capacity over the same period, were evaluated on an hourly basis for both onshore wind and solar PV. The spatial variation of factors impacting solar CF were modeled consistently, accounting for tilt, packing density, sun shading, and temperature. Hourly solar power values were calculated assuming installation of fixed-tilt polysilicon PV modules with a 16.2% conversion efficiency.

Onshore areas that are forested, urban, or covered with water or ice were filtered according to data from the NASA MODIS (Moderate Resolution Imaging Spectroradiometer) satellite MCD12C1 land cover product (NASA Land Processes Distributed Active Archive Center, 2012). Slope data were derived from the Shuttle Radar Topography Mission (SRTM) Global Enhanced Slope Database (Jarvis et al., 2008) with a spatial resolution of 1 arc-s (∼30 m). Grids characterized by slopes of more than 20% or by heights of more than 3000 m were excluded as inappropriate for deployment of onshore wind power systems.

Our study used slope, land use type, and solar radiation as criteria to identify areas suitable for solar farm development, following the approach described by Chen et al. (2019). The maximum permissible slope was set at 5%. As with onshore wind, the SRTM database was used to calculate terrain elevation and slopes for each grid. Suitability factors were selected according to land use types with higher values allocated to land areas with sparse vegetation and low ecological productivity (Noorollahi et al., 2016). The MODIS data were used to filter and eliminate unsuitable land areas, excluding forests, water bodies, permanent wetlands, croplands, cropland/natural vegetation mosaic, and snow and ice environments (land classifications are displayed in Figure S2). Areas excluded by these filters were assigned 0% as suitability factors. For exploitable areas, suitability factors ranging from 5 to 20% were assigned to each land use type. The minimum solar radiation required for exploitable land areas was set at 1400 kWh m^−2^ a^−1^, a typical threshold value for acceptable solar resources (Chen et al., 2019). And, it should be noted, the current study does not allow for a potential source of carbon-free electric power from solar panels installed on roof tops, a development that could be facilitated by appropriately targeted policy initiatives.

Onshore power was calculated using an inter turbine spacing of 9 rotor diameters (one turbine per 0.64 km^2^) assuming rated power for the turbines of 2.5 MW. The area for each latitude/longitude grid cell was divided in this manner to compute the number of turbines that could fit maximally into a given cell. The potential installed capacity (in GW) was computed by multiplying the number of turbines in a cell by the turbine power (2.5 MW in this case). The solar power PV capacity potential (in GW) is defined by the packing factor obtained by multiplying the power per unit area of the PV panels (161.9 W m^−2^) by the area available for their placement (factoring in solar filter constraints as described above). The spatialized packing factor here refers to the effective panel area per square meter of land area, which is determined by the solar PV tilt, azimuth angle (east-west orientation), and the spacing between neighboring PV panel footprints. The tilt setting assumed in the study follows the method proposed by Jacobson and Jadhav (2018), and the orientation of the panels was set to face the equator. The principle to determine the spacing between footprints is to ensure that minimal shading will occur for most of the sunlight hours throughout the year. The spacing was calculated using the solar altitude angle for 3 PM at the winter solstice, the day for which shading is likely to be most significant. Daily and hourly variations in the potential for wind and solar are indicated in Figure 1 for the national total and in Figure S1 for the five regions. While reanalysis data can overestimate the quality (and underestimate the variability) of the wind resource in parts of the world, we do note that the wind CFs used in our calculation are notably consistent with analysis conducted at the state-level by other India studies (Bhushan et al., 2014). We do acknowledge that reanalysis data may not perfectly represent fluctuations in the wind quality; however, our additional scenarios should account for the model’s sensitivity to lower wind output. Due to the computational demands associated with evaluating the optimal siting locations for renewables we calculate physical potentials for wind and solar at each of the five regions according to the spatial constraints indicated in Figure S2. That is to say, for the regional CFs, we computed the average CF across the region, acknowledging that this may actually underestimate the wind and solar generation potential relative to the real-world values.

The capital costs for solar and wind power in India in 2019 amounted to 618 $ kW^−1^ and 1055 $ kW^−1^, respectively (International Renewable Energy Agency, 2020). The cost reductions from 2020 to 2050 for the two technologies are taken as 49% and 39%, respectively, following the moderate trend defined in the NREL ATB database, yielding 2050 capital costs of 315 $ kW^−1^ for solar and 644 $ kW^−1^ for onshore wind (National Renewable Energy Laboratory, 2020).

#### Hydrogen technology options

Green hydrogen is considered an important alternative to fossil fuels in decarbonizing the planet. The future hydrogen economy aims not only to phase out the current hydrogen production from fossil fuels, but also to have hydrogen play important roles in the industrial, power generation and transport sectors. In the power sector, we explore the use of green hydrogen as a storage medium to address the inherent variability of solar and wind power generation. In the industrial sector, we consider applications of hydrogen as a reducing agent in the iron and steel sector, substituting for coking coal, a process referred to as hydrogen-DRI (direct reduced iron). We assume further that green hydrogen can be used as a feedstock for the production of ammonia and other chemicals and as a heat source in the manufacture of cement and other industrial commodities. We emphasize additionally the role it can play as an energy source for fuel cell vehicles and as an input for the production of (green) ammonia with applications for shipping and potentially also for other elements of India’s future transportation system.

We concluded that alkaline electrolyzer cells (AEC) can provide the most mature and durable option for production of the projected future demand for green hydrogen, estimated at an annual level of 137 Mt by 2050. Data on the capital cost and electrical efficiency for AEC were obtained from projections developed by the International Energy Agency (International Energy Agency, 2019). Geological reservoirs integrated with low-pressure compressors (20–120 bar) systems are proposed as an effective means for storage of gaseous hydrogen. Salt caverns have been exploited in a number of instances to meet this function both in Europe and in the US. The cost and other data employed here were adapted from a multi-year study published by the US Department of Energy (US DOE, 2016).

Stored hydrogen is used to produce power when wind and solar sources are temporally unable to keep up with demand. Hydrogen combustion is considered as the default option for hydrogen-based power generation, consistent with the net-zero roadmap identified by the IEA (Rastegar and Fotuhi-Firuzabad, 2015). An alternate approach could use fuel cells, the choice likely to depend on future developments in relative costs for the two technologies. The advantage of the direct combustion option is that it could make use of current investments in natural gas fired systems, transitioning gradually to use of increasing concentrations of hydrogen. The hydrogen combustion power generation option is assumed to have the same characteristics as an Indian gas plant. To avoid underestimating the impact of transportation costs on the hydrogen economy, an average delivery and distribution cost of 0.3 $ kg^−1^ has been included for all the generated hydrogen consistent with an estimate from US DOE (2016). The techno-economic characteristics of the hydrogen-related technologies are summarized in Table S10, with data referenced from previous studies (Buttler and Spliethoff, 2018; Finance, 2020; International Energy Agency, 2019; US DOE, 2016).

#### Least-cost energy system optimization model

The energy system configuration, hourly operation, related cost, and associated carbon emission for different scenarios were obtained based on a least-cost energy system optimization model (LCESOM), which optimizes jointly investment decisions and hourly system operations accounting for a full set of flexibility constraints. The model allows for potential deployment of defined renewable resources, for thermal generation, for hydrogen production, for energy storage, and for upgrades in interregional transmission. The decision variables for the LCESOM involve two components. For capacity investments, they account for invested capacities for each type of generation technology for each region, the capacity of storage deployed, and the capacity for transmission between different regions. For system operation, the decision variables allow for the available capacity and for the hourly dispatched output for each category of generation and storage for each region. The capacity available during the dispatch phase is interlinked with the investment decisions.

The objective of the LCESOM is to minimize the overall system cost, which includes two parts: system annual operational costs and amortized capacity investments. The model considers a full set of constraints for the system operation. Hourly electricity and hydrogen balance as well as reserve constraints are incorporated for each region. Flexibility constraints for thermal units are also included with maximum and minimum generation limits defined, and with specification of ramping and minimum on/off time constraints shown in Table S11 with data referenced from previous studies (Lu et al., 2020; Ministry of Power, 2019; Palchak et al., 2017; Rose et al., 2020; Sharma et al., 2018; Sinha, 2020). Operational constraints relating to energy storage are also considered based on different characteristics of storage technologies. Limitations on inter-regional power flow are incorporated in optimizing regional power exchange. Finally, renewable portfolio requirements are incorporated as an additional constraint.

To accelerate the calculation at such large scale, a novel flexibility method described by ref. (Han et al., 2019) is employed to reduce the modeling complexity and improve the computational efficiency. The units are grouped in the model with similar operational characteristics (same fuel type, similar nameplate capacity) to be dispatched based on aggregated power generation. There are 6 groups (categories) for each of the 5 regions in India and the total online capacity at each time interval is calculated allowing for a combination of on-off status for all individual units in the group. The mathematical formulation of the proposed optimization model is detailed as follows.

The decision variables in the thermal components are constrained by those in the capacity expansion component mainly in three ways. First, the hourly power generation and hourly online capacities are constrained by the total capacity available for a specific thermal technology (coal, gas, or hydrogen):

(Equation 1) $${\underline{\mu }}_{k}^{i}\cdot {\bar{p}}_{t,k}^{i}\leq p_{t,k}^{i}\leq {\bar{\mu }}_{k}^{i}\cdot {\bar{p}}_{t,k}^{i}\leq {\bar{I}}_{k}^{i}=I_{k}^{i}+I_{i,k}^{0}$$

where $p_{t,k}^{i}$ and ${\bar{p}}_{t,k}^{i}$ define the power generation and online capacity for the *i*^th^ category of thermal units in region *k* at time *t*; ${\underline{\mu }}_{k}^{i}$ and ${\bar{\mu }}_{k}^{i}$ are the minimum and maximum output ratios for the *i*^th^ category of thermal units in region k at hour t, respectively; ${\bar{I}}_{k}^{i}$, $I_{i,k}^{0}$ and $I_{k}^{i}$ denote the total capacity, the installed capacity and the newly expanded capacity (between 2019 and 2040 in this study) for the *i*^th^ category of thermal units in region *k*.

Wind and solar power are constrained by the total capacity, associated with the hourly capacity factor calculated based on physical potential:

(Equation 2) $$0\leq p_{t,k}^{w}\leq \alpha_{t,k}\cdot {\bar{I}}_{k}^{w}=\alpha_{t,k}\cdot \left(I_{k}^{w}+I_{w,k}^{0}\right)$$

(Equation 3) $$0\leq p_{t,k}^{s}\leq \beta_{t,k}\cdot {\bar{I}}_{k}^{s}=\beta_{t,k}\cdot \left(I_{k}^{s}+I_{s,k}^{0}\right)$$

where $p_{t,k}^{w}$ and $p_{t,k}^{s}$ represent the power generations for wind and solar in region *k* at time *t*. Hourly capacity factors for wind and solar are denoted by $\alpha_{t,k}$ and $\beta_{t,k}$. ${\bar{I}}_{k}^{x}$, $I_{k}^{x}$ and $I_{x,k}^{0}$ denote the total capacity, the installed capacity and the newly expanded capacity for wind (*x* = *w*) and solar (*x=s*) in region *k*.

The hourly power exchange between two regions is constrained by the inter-regional transmission capacity:

(Equation 4) $$-L_{j,k}-L_{j,k}^{0}={\bar{L}}_{j,k}\leq p_{j,k}^{t}\leq {\bar{L}}_{j,k}=L_{j,k}+L_{j,k}^{0}$$

where $p_{j,k}^{t}$ represents the transmitted power between regions *j* and *k* at hour *t*. ${\bar{L}}_{j,k}$ and ${\bar{L}}_{j,k}$ denote the maximum and minimum transmission capacities, respectively. $L_{j,k}$ and $L_{j,k}^{0}$ denote the installed capacity and the newly expanded capacity for inter-regional transmission corridor *j-k*.

The objective of the LCESOM is to minimize the overall system costs, which include two parts: annual operational costs $C_{op}$ with hourly resolution (fuel costs $C_{fuel}$, start-up costs $C_{st}$ and operational costs for the electricity storage system $C_{es}$, hydro power $C_{h}$ and nuclear power $C_{nu}$), and capacity costs $C_{cap}$ (amortized investment costs $C_{inv}$ and fixed O&M costs $C_{om}$):

(Equation 5) $$\min\;C_{op}+C_{cap}=\left(C_{fuel}+C_{st}+C_{es}+C_{h}+C_{nu}\right)+\left(C_{inv}+C_{om}\right)$$

such that:

(Equation 6) $$\left\{ \begin{array}{l} C_{inv}=\sum_{k=1}^{N_{a}}\left(\begin{array}{l} \overset{M_{k}}{\underset{i=1}{\sum }}a_{k}^{i}\cdot {\bar{I}}_{k}^{i}+a_{k}^{w}\cdot {\bar{I}}_{k}^{w}+a_{k}^{s}\cdot {\bar{I}}_{k}^{s}+a_{k}^{h}\cdot {\bar{I}}_{k}^{h}+a_{k}^{nu}\cdot {\bar{I}}_{k}^{nu}\\ +a_{k}^{ec}\cdot {\bar{I}}_{k}^{\;ec}+a_{k}^{c}\cdot {\bar{I}}_{k}^{c}+a_{k}^{hs}\cdot {\bar{I}}_{k}^{hs}\\ +\sum_{z=1}^{N_{es}}\left(a_{k}^{p,z}\cdot {\bar{I}}_{k}^{\;p,z}+a_{k}^{e,z}\cdot {\bar{I}}_{k}^{\;e,z}\right)+\sum_{(j,k)\in \Psi }a_{j,k}^{l}\cdot {\bar{L}}_{j,k} \end{array}\right)\\ C_{om}=\sum_{k=1}^{N_{a}}\left(\begin{array}{l} \overset{M_{k}}{\underset{i=1}{\sum }}f_{k}^{i}\cdot {\bar{I}}_{k}^{i}+f_{k}^{w}\cdot {\bar{I}}_{k}^{w}+f_{k}^{s}\cdot {\bar{I}}_{k}^{s}+f_{k}^{ec}\cdot {\bar{I}}_{k}^{\;ec}+f_{k}^{c}\cdot {\bar{I}}_{k}^{c}\\ +f_{k}^{hs}\cdot {\bar{I}}_{k}^{hs}+\sum_{(j,k)\in \Psi }f_{j,k}^{l}\cdot {\bar{L}}_{j,k} \end{array}\right) \end{array}\right.$$

(Equation 7) $$\left\{ \begin{array}{l} C_{fuel}=\overset{N_{a}}{\underset{k=1}{\sum }}\overset{M_{k}}{\underset{i=1}{\sum }}\overset{T}{\underset{t=1}{\sum }}c_{k}^{i}\cdot p_{t,k}^{i}\\ C_{st}=\overset{N_{a}}{\underset{k=1}{\sum }}\overset{M_{k}}{\underset{i=1}{\sum }}\overset{T}{\underset{t=1}{\sum }}SD_{k}^{i}\cdot s_{t,k}^{i}\\ C_{es}=\overset{N_{a}}{\underset{k=1}{\sum }}\overset{N_{es}}{\underset{z=1}{\sum }}\overset{T}{\underset{t=1}{\sum }}c_{k}^{es,z}\cdot \left(p_{t,k}^{dis,z}+p_{t,k}^{ch,z}\right)\\ C_{h}=\overset{N_{a}}{\underset{k=1}{\sum }}\overset{T}{\underset{t=1}{\sum }}c_{k}^{h}\cdot p_{t,k}^{h}\\ C_{nu}=\overset{N_{a}}{\underset{k=1}{\sum }}\overset{T}{\underset{t=1}{\sum }}c_{k}^{nu}\cdot p_{t,k}^{nu} \end{array}\right.$$

where *N*_*a*_ and *M*_*k*_ denote the numbers of regions and categories of thermal units in region *k*; $a_{k}^{i}$ and $f_{k}^{i}$ are the amortized investment costs and fixed O&M costs for thermal units; $a_{k}^{x}$ and $f_{k}^{x}$ are the amortized investment costs and fixed O&M costs for wind power (*x=w*), solar power (*x=s*), hydro power (*x=h*), nuclear power (*x=nu*), electrolyzers (*x=ec*), compressors (*x=c*) and hydrogen storage (*x=hs*); ${\bar{I}}_{k}^{h}$ and ${\bar{I}}_{k}^{nu}$ are the installed capacity for hydro and nuclear power systems; ${\bar{I}}_{k}^{\;p,z}$ and ${\bar{I}}_{k}^{\;e,z}$ denote the power and energy capacities for the newly installed electricity storage systems; ${\bar{I}}_{k}^{ec}$, ${\bar{I}}_{k}^{c}$ and ${\bar{I}}_{k}^{hs}$ denote capacities for the newly installed electrolyzers, compressors, and hydrogen storage systems; $a_{k}^{p,z}$ and $a_{k}^{e,z}$ represent the corresponding power-specific and energy-specific amortized investment costs for *z*^th^ category of electricity storage in region *k*. $a_{j,k}^{l}$ is the investment cost for the newly expanded capacity for inter-regional transmission corridor. Ψ denotes the set of the inter-regional transmission corridors. The operational costs for thermal units, energy storage, hydro power and nuclear power are $c_{k}^{i}$, $c_{k}^{es,z}$, $c_{k}^{h}$ and $c_{k}^{nu}$. $SD_{k}^{i}$ represents the start-up cost for thermal units.

Constraints including unit commitment, operations for energy storages and hydrogen production, system power balances and reserves, renewable penetration and intra-regional power flow are considered in the model. The unit commitment of thermal plants including all of the flexibility constraints are detailed in our previous publications (Chen et al., 2018; Han et al., 2019).

Constraints relating to operations for energy storage are considered based on different regional distributions and storage types. The charging ($p_{t,k}^{ch,z}$) and discharging ($p_{t,k}^{dis,z}$) power for the *z*^th^ category of electricity storage in region *k* is constrained by the non-negative power capacity of newly installed storage; The hydrogen storage charging ($h_{t,\;k}^{ch}$) and discharging ($h_{t,\;k}^{dis}$) are constrained by the installed capacity of supporting compressors.:

(Equation 8) $$0\leq p_{t,k}^{dis,z}\leq {\bar{I}}_{k}^{\;p,z}$$

(Equation 9) $$0\leq p_{t,k}^{ch,z}\leq {\bar{I}}_{k}^{\;p,z}$$

(Equation 10) $$0\;\leq \;h_{t,\;k}^{ch}\;\leq {\bar{I}}_{k}^{c}$$

(Equation 11) $$0\;\leq \;h_{t,\;k}^{dis}\;\leq {\bar{I}}_{k}^{c}$$

The energy flow from a storage system is restricted by the following energy balance:

(Equation 12) $$e_{t+1,k}^{es,z}=e_{t,k}^{es,z}+\gamma_{es}^{ch,z}\cdot p_{t,k}^{ch,z}-\frac{1}{\gamma_{es}^{dis,z}}\cdot p_{t,k}^{dis,z}-\gamma_{es}^{self,z}\cdot e_{t,k}^{es,z}$$

(Equation 13) $$e_{t+1,k}^{hs}=e_{t,k}^{hs}+\gamma_{hs}^{ch}\frac{1}{2}h_{t,\;k}^{ch}-\frac{h_{t,\;k}^{dis}}{\gamma_{hs}^{dis}}-\gamma_{hs}^{self}\cdot e_{t,k}^{hs}$$

where $e_{t,k}^{es,z}$ denotes the energy state that satisfies $0\leq e_{t,k}^{es,z}\leq {\bar{I}}_{k}^{\;e,z}$ and $0\;\leq \;e_{t,k}^{hs}\;\leq {\bar{I}}_{k}^{\;hs}$. The energy state in the last time interval is equal to that of the first time interval. ($\gamma_{es}^{ch,z}$, $\gamma_{es}^{dis,z}$, $\gamma_{hs}^{ch}$, $\gamma_{hs}^{dis}$) and ($\gamma_{es}^{self,z}$, $\gamma_{hs}^{self}$) represent the energy efficiency and loss (self-discharge) rates.

To quantify the spinning reserve $r_{t,k}^{es,z}$ provided by storage systems, the following formulation is considered:

(Equation 14) $$0\leq p_{t,k}^{dis,z}+r_{t,k}^{es,z}\leq {\bar{I}}_{k}^{p,z}$$

(Equation 15) $$0\leq r_{t,k}^{es,z}\leq {\bar{I}}_{k}^{\;p,z}$$

(Equation 16) $$0\leq e_{t,k}^{es,z}-\frac{1}{\gamma_{es}^{dis,z}}\cdot \left(p_{t,k}^{dis,z}+r_{t,k}^{es,z}\right)-\gamma_{es}^{self,z}\cdot e_{t,k}^{es,z}$$

Electrolyzer and compressor operation are constrained by their capacities based on different regional distributions:

(Equation 17) $$0\leq p_{t,k}^{ec}\leq {\bar{I}}_{k}^{ec}$$

(Equation 18) $$0\leq h_{t,\;k}^{ch}+h_{t,\;k}^{dis}\leq {\bar{I}}_{k}^{c}$$

(Equation 19) $$h_{t,\;k}^{ec}=p_{t,k}^{ec}\cdot \gamma_{ec}$$

(Equation 20) $$p_{t,k}^{c}=\gamma_{c}\cdot \left(h_{t,\;k}^{ch}+h_{t,\;k}^{dis}\right)$$

where $p_{t,k}^{ec}$, $h_{t,\;k}^{ec}$ and $\gamma_{ec}$ are the consumed power, hydrogen output and power to hydrogen efficiency for the electrolyzers; $\;p_{t,k}^{c}$ and $\gamma_{c}$ indicate the consumed power and compression energy cost for the compressors.

Constraints for the power balance and reserve are considered to meet requirements for the reliability of power grid operation. At each time step, the power demand plus electrolyzer and compressor consumption equals the sum of the power outputs from all thermal units, hydropower and nuclear power plants, electricity storage systems, wind, solar and inter-regionally transmitted power. The hydrogen demand is equal to the sum of the hydrogen outputs from the electrolyzers and hydrogen storage:

(Equation 21) $$\sum_{i=1}^{M_{k}}p_{t,k}^{i}+p_{t,k}^{w}+p_{t,k}^{s}+p_{t,k}^{h}+p_{t,k}^{nu}+\sum_{j\in \Psi_{k}}p_{j,k}^{t}+\sum_{z=1}^{N_{es}}\left(p_{t,k}^{dis,z}-p_{t,k}^{ch,z}\right)=D_{t,k}+p_{t,k}^{ec}+p_{t,k}^{c}$$

(Equation 22) $$h_{t,\;k}^{ec}+h_{t,\;k}^{dis}=h_{t,\;k}^{ch}+\sum_{i=4}^{M_{k}}h_{t,\;k}^{i}$$

where $\Psi_{k}$ defines the set of regions connected to region *k*. *D*_*t,k*_ and $H_{t,k}$ denotes the power and hydrogen demand at time *t* for region *k*; $p_{t,k}^{h}$ and $p_{t,k}^{nu}$ represent the hydropower and nuclear power; $h_{t,\;k}^{i}$ indicates the hydrogen consumed by the *i*^th^ category of hydrogen turbines at time *t* for region *k*.

The forecasting errors in energy demand $R^{d}$ and wind $R^{w}$ and solar $R^{s}$ output are considered in the following reserve constraints:

(Equation 23) $$\sum_{i=1}^{M_{k}}{\bar{\mu }}_{k}^{i}\cdot {\bar{p}}_{t,k}^{i}+\alpha_{t,k}\cdot {\bar{I}}_{k}^{w}+\beta_{t,k}\cdot {\bar{I}}_{k}^{s}+\gamma_{t,k}\cdot {\bar{I}}_{k}^{h}+{\bar{I}}_{k}^{nu}+\sum_{z=1}^{N_{es}}\left(p_{t,k}^{dis,z}+r_{t,k}^{es,z}\right)+\sum_{j\in \Psi_{k}}p_{j,k}^{t}\geq \left(1+R^{d}\right)\cdot D_{t,k}+R^{w}\cdot p_{t,k}^{w}+R^{s}\cdot p_{t,k}^{s}$$

The constraints of downward reserve are not considered in the model because wind and solar can provide the downward reserve by curtailing their power output in real time during contingency, and thus the downward reserve is not binding in day-ahead scheduling. The minimum load (over generation) problem is captured mainly by the minimum power output and ramping constraints in this formulation.

To meet requirements for system reliability, the system invested capacity must be no less than the required capacity standard:

(Equation 24) $$\sum_{i=1}^{M_{k}}{\bar{I}}_{k}^{i}+\lambda_{k}^{w}\cdot {\bar{I}}_{k}^{w}+\lambda_{k}^{s}\cdot {\bar{I}}_{k}^{s}+\sum_{z=1}^{N_{es}}\left(\lambda_{k}^{es,z}\cdot {\bar{I}}_{k}^{p,z}\right)+\lambda_{k}^{h}\cdot {\bar{I}}_{k}^{h}+{\bar{I}}_{k}^{u}\geq D_{k}^{max}$$

where $D_{k}^{max}$ is the required capacity to satisfy reliability standards; $\lambda_{k}^{w}$, $\lambda_{k}^{s}$, $\lambda_{k}^{h}$ and $\lambda_{k}^{es,z}$ denote the capacity credits for wind, solar, hydro power and electricity storage in region *k*.

Constraints for renewable portfolio requirements are incorporated to set different levels of renewable penetration for the energy system:

(Equation 25) $$\overset{N_{a}}{\underset{k=1}{\sum }}\overset{T}{\underset{t=1}{\sum }}\left(p_{t,k}^{w}+p_{t,k}^{s}\right)-\overset{N_{a}}{\underset{k=1}{\sum }}\overset{N_{es}}{\underset{z=1}{\sum }}\overset{T}{\underset{t=1}{\sum }}\left(p_{t,k}^{ch,z}-p_{t,k}^{dis,z}\right)=\Gamma \cdot \overset{N_{a}}{\underset{k=1}{\sum }}D_{t,k}$$

where Γ (40≤Γ≤80% in the second scenario of this study) is the required renewable percentage for the overall renewable portfolio target.

The above optimization model minimizes the overall costs for power grid operation and capacity investment under a specified renewable penetration, as indicated in Equations (5), (6), and (7), constrained by Equations (1), (2), (3), and (4) associated with investment and operational decisions, unit commitment, (8)‒(16) for storage operation, (17)‒(20) for electrolyzer operation, (21)‒(24) for system energy balance and reserves, and (25) for the required percentage of renewables.

The amortized investment cost $a_{x}\;$ and fixed O&M cost $f_{x}$ for technology x are computed using the following equations:

(Equation 26) $$a_{x}=c_{x}\cdot \frac{r\cdot {\left(1+r\right)}^{l_{x}}}{{\left(1+r\right)}^{l_{x}}-1}$$

(Equation 27) $$f_{x}=c_{x}\cdot om_{x}$$

where $c_{x}$ is the capital cost of technology x; r is the interest rate, which is assumed to be 7% in this study; $l_{x}$ is the lifespan and $om_{x}$ is the annual operational and maintenance cost as in percentage of capital cost.

## Data Availability

Wind and solar data are publicly available from the NASA’s Goddard Earth Observing System. All other data are available in the manuscript or the supplemental information. Code for the model can be made available upon request.
