# Supplementary material for: Deep decarbonization of the Indian economy: 2050 prospects for wind, solar, and green hydrogen
Source: iScience. 2022 May 13;25(6):104399. doi: 10.1016/j.isci.2022.104399 (PMC9136670; doi:10.1016/j.isci.2022.104399)
Supplement: Document S1. Tables S1–S10 and Figures S1–S6 [file mmc1.pdf]

iScience, Volume *25*

Supplemental information

Deep decarbonization of the Indian economy: 2050 prospects for wind, solar, and green hydrogen

Shaojie Song, Haiyang Lin, Peter Sherman, Xi Yang, Shi Chen, Xi Lu, Tianguang Lu, Xinyu Chen, and Michael B. McElroy

Table S1. Key socioeconomic assumptions for India, Related to STAR Methods.

	Unit	2020	2030	2040	2050
GDP	Trillion $	2.9	5.3	9.5	16.0
GDP growth rate	%	6.0	6.5	5.7	5.0
Population	Billion	1.38	1.50	1.59	1.64
Urbanization	%	37	42	46	50

Table S2. Projected road transport vehicles stock (in units of million vehicles) over the coming decades, Related to STAR Methods.

	2015	2020	2025	2030	2035	2040	2045	2050
**2-Wheelers**	169	198	233	294	372	471	635	856
**3-Wheelers**	6	9	12	17	24	35	48	66
**4-Wheelers**	36	47	61	76	95	119	169	241
**Buses**	3	3	3	3	4	4	6	7
**Trucks**	4	7	12	15	18	22	25	28

Table S3. Projected oil, electricity and hydrogen demands in the road transport sector. It is assumed here, in 2050, that the 2-/3-/4-wheelers use electric power, and that the buses and trucks use either oil or hydrogen, Related to STAR Methods.

	2015	2050		
	**Oil (PJ)**	**Electricity (TWh)**	**Oil (PJ)**	**Hydrogen (Mt)**
**2-Wheelers**	815	224	0	0
**3-Wheelers**	370	289	0	0
**4-Wheelers**	897	508	0	0
**Buses**	745	0	1909	6.8
**Trucks**	1103	0	6306	24.9
**Total**	**3931**	**1021**	**8215**	**31.7**

Table S4. Final energy service demand prediction in the residential, commercial and agriculture sectors (Units: PJ), Related to STAR Methods.

	2015	2020	2025	2030	2035	2040	2045	2050
**Residential**	3989	4293	4747	5422	6418	7787	9638	12189
**Commercial**	635	777	1002	1236	1541	1924	2391	2973
**Agriculture**	1037	1074	1113	1154	1375	1638	1952	2326

Table S5. Key industrial product demand projections in India (Units: Mt), Related to STAR Methods.

	2015	2020	2025	2030	2035	2040	2045	2050
**Cement**	283.5	349.0	483.0	710.0	815.1	935.8	1074.4	1250.0
**Caustic Soda**	2.5	5.0	6.7	9.0	10.8	12.6	14.3	16.3
**Soda**	2.6	5.1	6.8	9.1	10.8	12.7	14.4	16.4
**Fertilizer**	41.3	43.6	46.3	48.9	51.6	53.5	55.7	57.9
**Iron & Steel**	95.5	126.6	167.8	222.5	251.7	284.8	322.2	364.6
**Aluminum**	2.4	2.9	3.9	5.2	5.9	6.6	7.5	8.5
**Paper**	17.0	22.5	29.9	39.6	43.7	48.3	53.3	58.8
**Textile**	68.4	79.3	91.9	106.6	117.0	128.6	141.2	155.3

Table S6. Annual national level electricity consumption by sectors (Units: TWh), Related to STAR Methods.

	2015	2050
**Industry**	395	2103
**Residential**	239	3386
**Commercial**	119	826
**Transport**	22	1361
**Agriculture**	173	646
**Total**	947	8323

Table S7. Per unit expansion costs associated with interregional transmission of power in 2020. The number and location of current corridors between different regions are assumed to be fixed but the transmission capacity could be expanded. Per unit expansion cost is defined as the ratio of total investment cost for a corridor to its capacity, Related to STAR Methods.

Interregional corridor	Cost ($ kW^{-1})
East-North	88.75
East-Northeast	65.46
East-South	95.42
East-West	64.55
North-West	88.71
North-Northeast	329.81
South-West	58.16

Table S8. Properties of electricity storage technologies in 2020, Related to STAR Methods.

	Battery	Pumped Hydro
**Round-trip efficiency (%)**	86	80
**Energy loss rate ($hour^{-1}$)**	0.001	0
**Energy-specific cost ($ kWh^{-1})**	299	70
**Power-specific cost ($ kW^{-1})**	259	1400
**Operational cost ($ MWh^{-1})**	3.5	6
**Lifetime (year)**	15	50

Table S9. Specifications for the onshore wind turbine used in the analysis, Related to STAR Methods.

Specifications	GE 2.5 MW
Rated power	2500 kW
Cut-in wind speed	3.0 m/s
Rated wind speed	13.0 m/s
Cut-out wind speed	25.0 m/s
Diameter	100.0 m
Swept area	7853.9 m^2
Number of blades	3

Table S10. Properties of hydrogen related technologies in 2050, Related to STAR Methods.

	AEC	Compression	Salt Cavern[b]
**Capital cost**	200 $ kW^{-1}	0.36 k$ kg^{-1} h^{-1}	4.5 $ kg^{-1}
**Electrical efficiency (%, LHV[a])**	70	/	/
**Electricity demand (kWh kg^{-1})**	/	0.84	/
**Loss (%)**	/	0.5	0
**Annual O&M cost (% of capital cost)**	2	2	2
**System lifetime (year)**	30	15	30

Notes: All the parameters are hydrogen or hydrogen equivalent based values.

[a] LHV = lower heating value.

[b] The cost is levelized for a 1100-ton geologic storage system.

Table S11. Properties of small (10-300 MW), medium (300-600 MW), and large (600-1000 MW) thermal (coal and natural gas) units in 2020, Related to STAR Methods.

	Coal			Gas		
	**Small**	**Medium**	**Large**	**Small**	**Medium**	**Large**
**Max. output (%)**	100	100	100	100	100	100
**Min. output (%)**	55	55	55	20	50	50
**Capital cost ($ kW^{-1})**	1034	974	857	756	704	603
**O&M (% of Capital cost)**	2.1	2.0	1.9	2.6	2.5	2.4
**Start-up cost ($ MW^{-1})**	234	234	212	105	105	105
**Min. up time (hour)**	8	24	24	2	4	8
**Min. down time (hour)**	8	24	48	2	8	12
**Ramping limit (% hr^{-1})**	50	50	50	100	100	50
**Fuel use (kcal kWh^{-1})**	2747	2622	2274	2075	2031	2031
**Lifetime (year)**	40	40	40	30	30	30
**CO_2 emission (t MWh^{-1})**	1.05	1.00	0.87	0.43	0.42	0.42

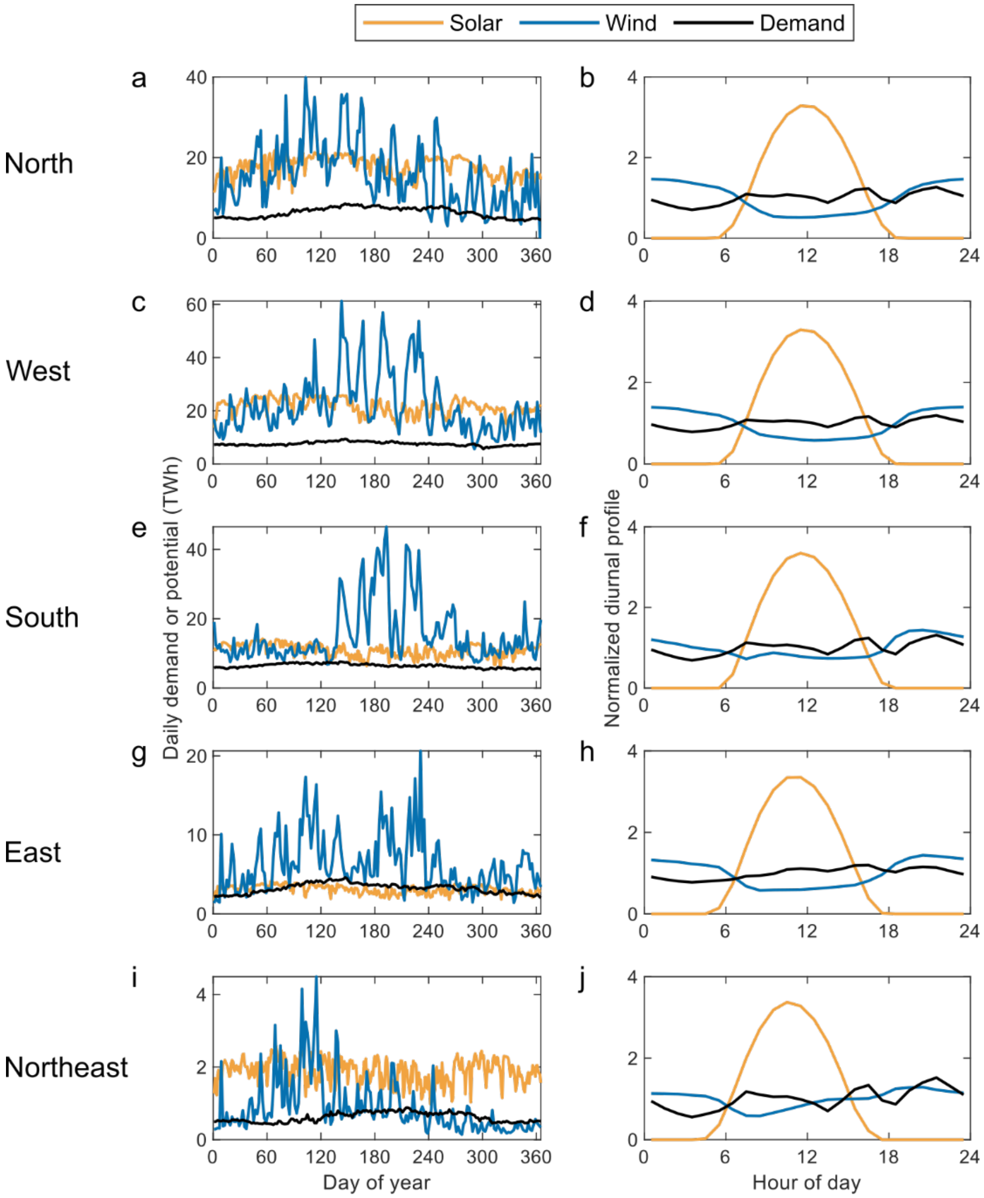

Figure S1. Solar and wind power potential and 2050 demand for the five regions (North, West, South, East, and Northeast), Related to Figure 1. The left-side panels (a, c, e, g, and i) show daily variability and the right-side panels (b, d, f, h, and j) indicate diurnal profiles normalized by the 24-hour average values (y-axis unit free).

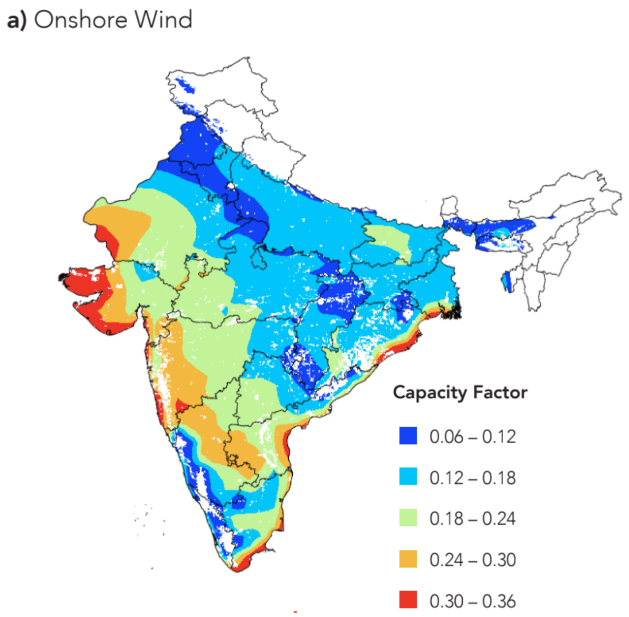

Figure S2. Renewable capacity factors, Related to STAR Methods. Spatial distributions of mean annual capacity factors for **a** on- and offshore wind and **b** solar PV constructed based on meteorological output from the MERRA-2 reanalysis product.

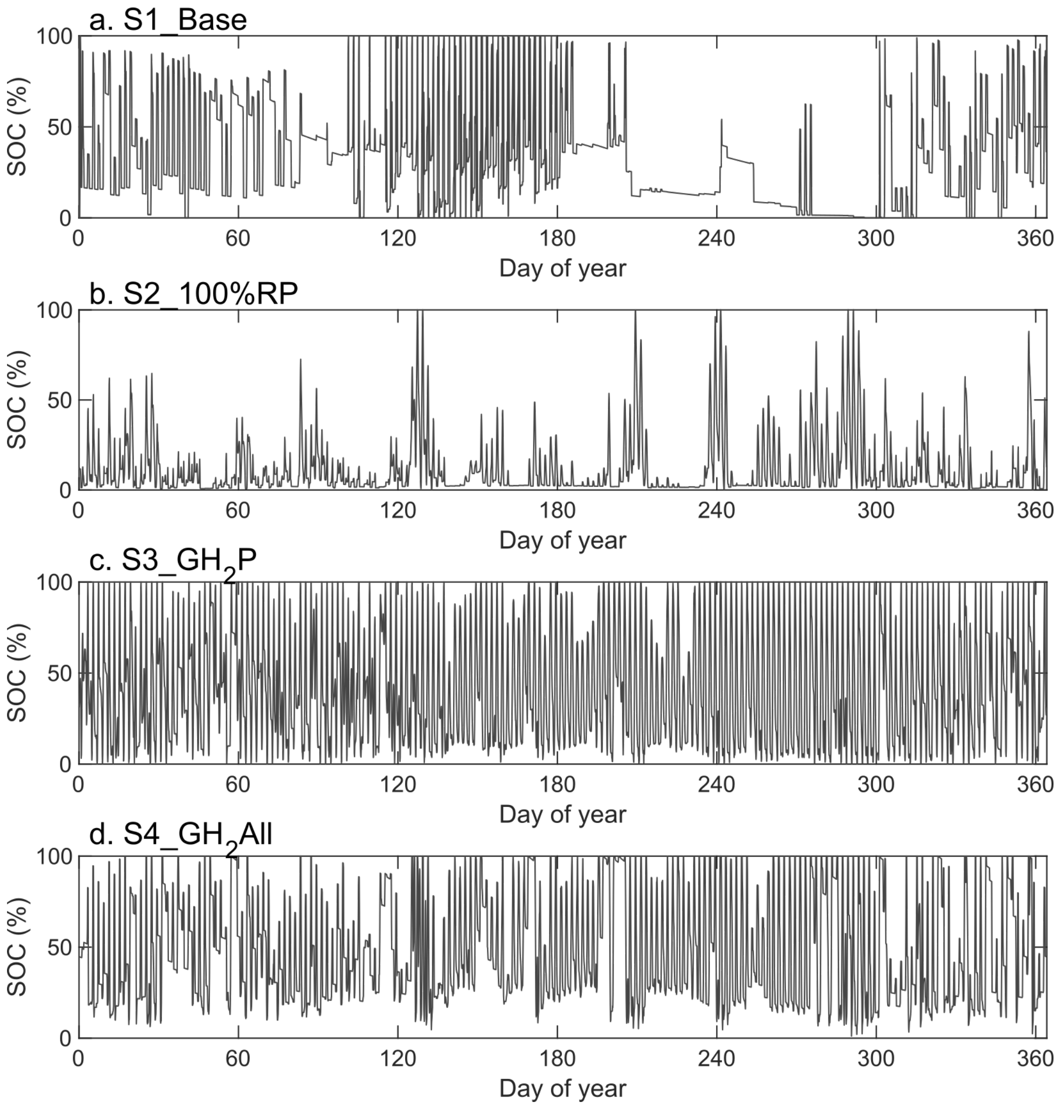

Figure S3. Hourly variability of state of charge (SOC) for electricity storage, Related to Results and Discussion. Country-wide hourly SOC for electricity storage identified for the selected 4 cases. The SOC is storage's available capacity expressed as a percentage of its installed capacity.

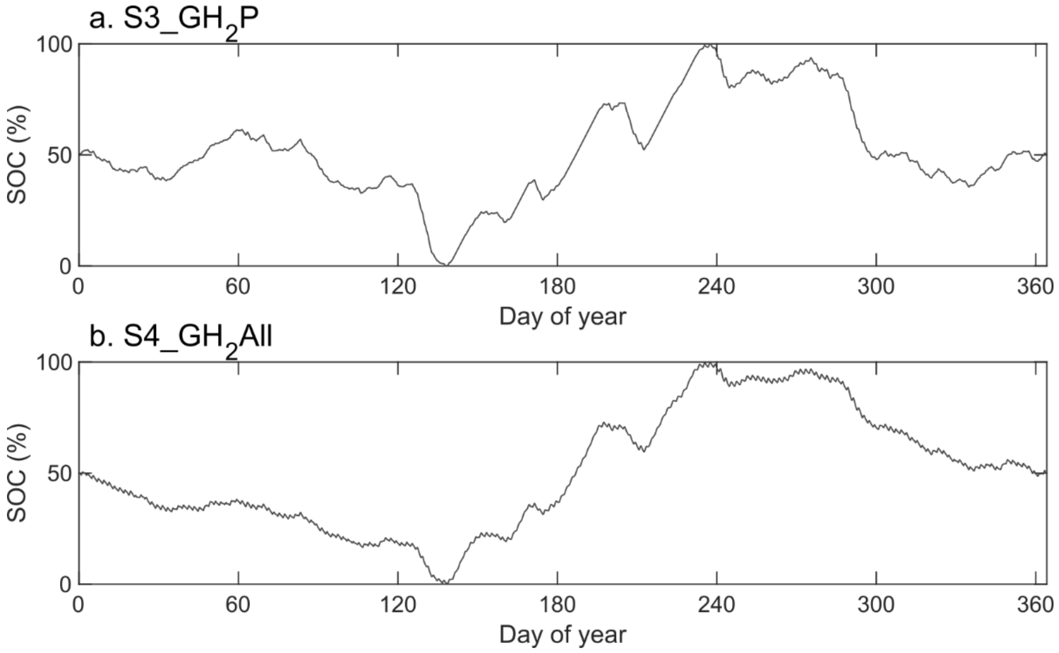

Figure S4. Hourly variability of state of charge (SOC) for hydrogen storage, Related to Results and Discussion. Country-wide hourly SOC for hydrogen storage identified for the selected two cases have hydrogen application (a. S3_GH$_2$P, b. S4_GH$_2$All). The SOC is storage's available capacity expressed as a percentage of its installed capacity.

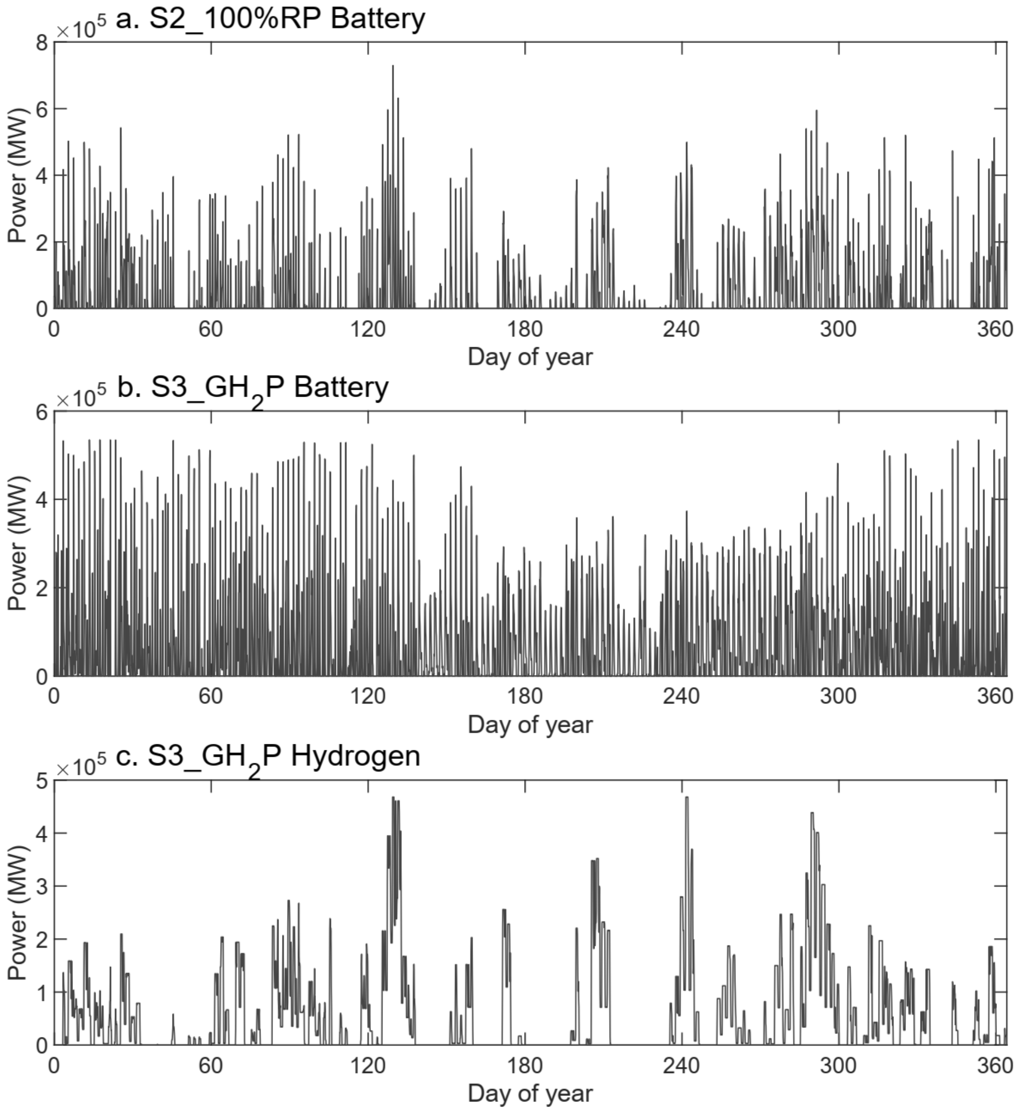

Figure S5. Country-wide hourly variability of power generation from battery and hydrogen system, Related to Results and Discussion.

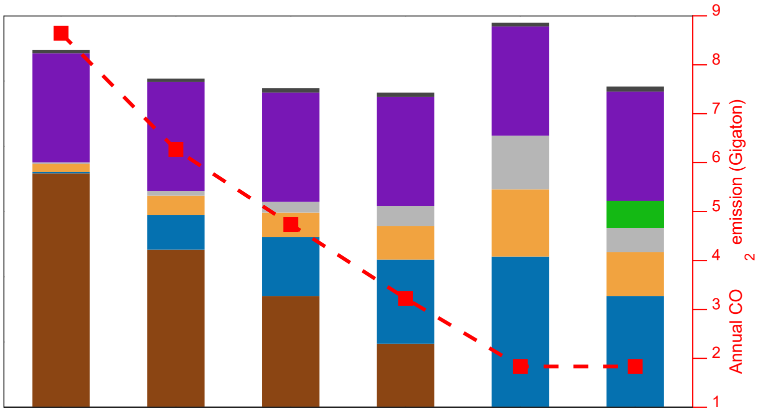

Figure S6. National energy supply cost and related CO_2 emissions, Related to Figure 5. Thermal power refers to the costs for fuel, amortized capital, operational and maintenance expenses for coal and gas-fired plants. Hydrogen related refers to the costs associated with capital and operational expenses for electrolyzers, hydrogen turbine, compressor, and hydrogen storage. Fossil fuel other refers to the costs for coal, oil and natural gas consumed by selected sectors excluding power generation. Others refer to the costs for amortized capital and operational and maintenance expenses for hydro and nuclear plants.

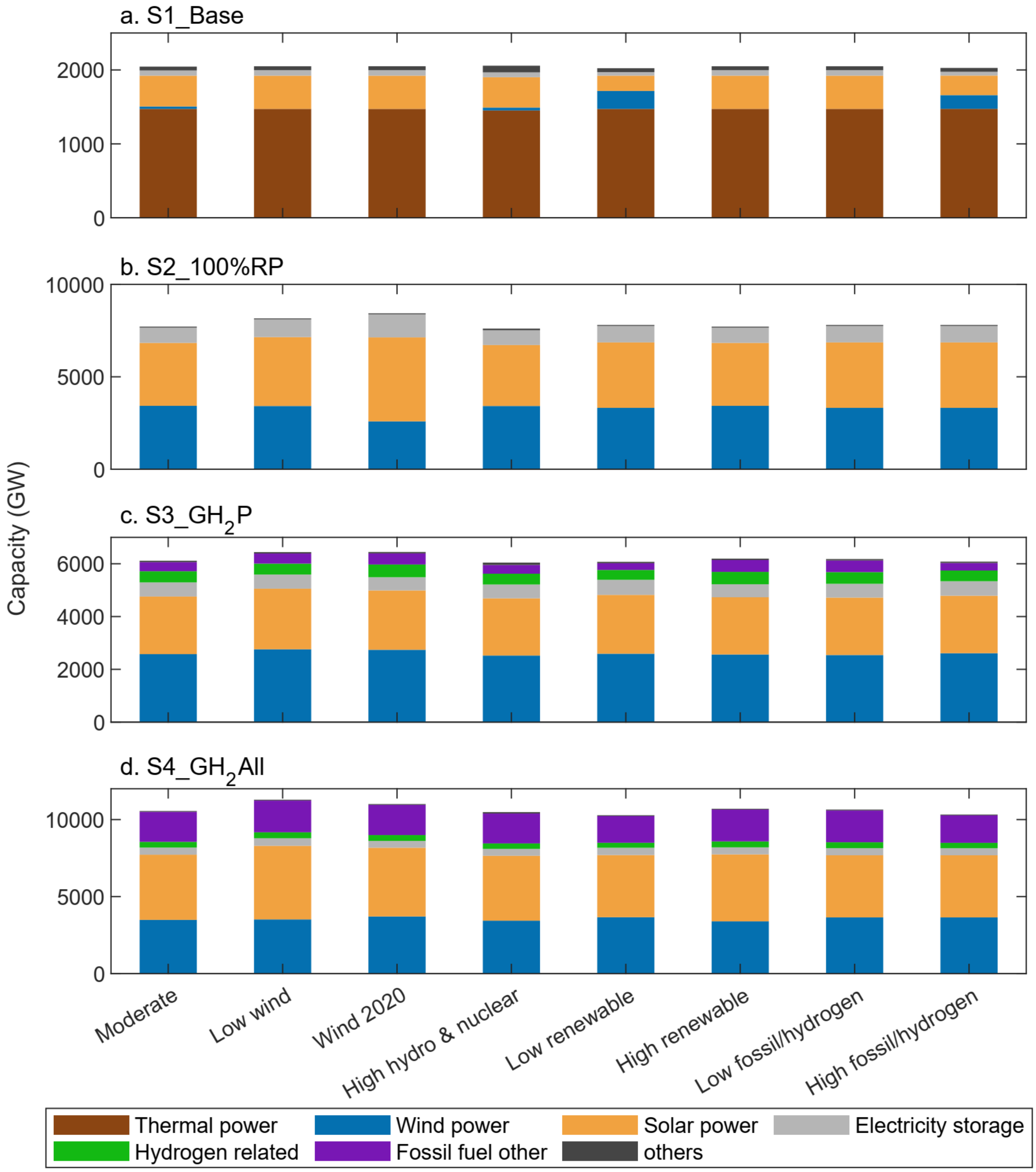

Figure S7. National installed capacities in various scenarios, Related to Figure 4. Country-wide installed capacities (GW) for different technologies identified using the optimization model with parameters appropriate for all the sensitivity scenarios. Others represent the capacities for hydro and nuclear power.
